# Use of Upadacitinib in elderly patients with IBD: a case series

**DOI:** 10.1186/s40780-026-00541-x

**Published:** 2026-01-16

**Authors:** Ruby Arai, Kazuyo Okayama, Takanori Nishiguchi, Masayuki Fukata

**Affiliations:** Center for Inflammatory Bowel Disease, Department of Internal Medicine, Tokyo Yamate Medical Center, Japan Community Healthcare Organization, 3-22-1 Hyakunin-cho, Shinjuku-ku, Tokyo, 169-0073 Japan

**Keywords:** Inflammatory bowel disease, Upadacitinib, Elderly, Case series

## Abstract

**Background:**

Upadacitinib (UPA) is a selective Janus kinase (JAK) 1 inhibitor that may rapidly induce and maintain a remission of inflammatory bowel disease (IBD) through potent suppression of cytokine signaling. While a pan-JAK inhibitor has been shown to increase the risk of thromboembolic and major cardiovascular events (MACE), especially in elderly patients with rheumatoid arthritis, UPA may potentially be a better therapeutic option for elderly IBD patients owing to its selective effect on JAK1. However, the usefulness of UPA in elderly IBD patients has yet to be well assessed.

**Methods:**

To explore the efficacy and safety of UPA in elderly IBD patients, we conducted a retrospective case series study of patients with ulcerative colitis (UC) and Crohn’s disease (CD) aged over 60 years who received UPA from December 2022 to March 2025. Clinical response and remission rates, incidence of adverse events, and clinical outcomes were analyzed.

**Results:**

A total of 8 patients (5 UC and 3 CD) were included in this study. The rate of clinical response in UC was 40% at week 8, 80% at week 16, and the remission rate was 60% at week 16. One patient with acute severe UC underwent colectomy at week 3 due to resistance to UPA, although the patient had also failed a rescue therapy with infliximab (IFX). In patients with CD, the rate of clinical response and remission at week 12 was 33.3% each. After 52 weeks of treatment, 60% of UC and 66.7% of CD patients had sustained UPA treatment. During the median follow-up time of 14.1 months (95% CI 9.2, 24.4 months), adverse events of Common Terminology Criteria for Adverse Events (CTCAE) grade 2 or higher were identified in two cases. Although one patient with UC discontinued UPA at week 36 due to anemia and liver damage, most adverse events observed were otherwise mild. No serious infections, thromboembolic events, MACE, or malignancies were noted.

**Conclusions:**

While the small sample size precludes definitive conclusions, this case series suggests that UPA has potential efficacy and relatively acceptable tolerability in elderly patients with IBD.

## Background

The global increase in the incidence of IBD is leading to a growing number of elderly IBD patients. Therefore, it is estimated that the proportion of elderly IBD patients will be projected to reach approximately 30% by 2030 [1]. This demographic shift is attributed to an increase of newly diagnosed elderly individuals as well as the aging of IBD patients who have been diagnosed at a younger age [2]. While treatments of IBD often involve immunosuppression and the levels of which depend on the severity of disease, patients with elderly IBD tend to be at a higher risk of adverse events compared to younger patients due to age-related physiological changes and comorbidities [3]. Therefore, achieving a therapeutic balance between efficacy and safety in this population remains a clinical challenge.

The treatment of IBD has greatly advanced over the last two decades, particularly with the development of biologic agents. The use of biologics has led to a significant reduction of relapses, steroid dependency, and the rate of surgery in IBD [4]. Recent application of orally available small molecules has brought additional advancement, and the JAK inhibitors, one of the small molecule agents, have a rapid onset of action with their ability to simultaneously block multiple cytokine signaling pathways. The pairs of four JAKs, including JAK1, JAK2, JAK3, and Tyk2, induce intracellular cytokine signaling, and JAK1-mediated signaling has been associated with the pathology in IBD [5]. Among currently available JAK inhibitors for IBD, UPA is known to selectively inhibit JAK1 and has demonstrated efficacy for both UC and CD [6, 7]. Another JAK inhibitor that inhibits broad JAK activation (JAK 1–3) has been associated with an increased risk of MACE and malignancy in patients with rheumatoid arthritis [6]. Although UPA may have fewer off-target effects than the pan-JAK inhibitor, careful monitoring for adverse events would still be crucial in elderly IBD patients, as UPA is used at higher doses in IBD than in other conditions [7]. However, there is currently a paucity of data regarding the efficacy and safety of UPA in elderly patients with IBD. Here, we report the clinical responses, safety, and treatment outcomes of UPA in elderly patients with IBD.

## Methods

### Study design and subjects

We conducted a case series study by retrospectively collecting the clinical and laboratory data of consecutive patients with UC and CD who were over 60 years old and had received UPA for induction of remission at Tokyo Yamate Medical Center between December 2022 and March 2025, with a follow-up until September 2025 using the electronic medical record. Patients were identified by the key terms “Crohn’s disease” and “Ulcerative colitis” and stratified by age of 60 and older in the electronic medical records, followed by searching for Upadacitinib in the prescription database. The diagnoses of UC and CD were confirmed based on clinical manifestations, endoscopic findings, pathology results of mucosal biopsies, and the clinical course of the disease, with reference to the practice guidelines [8]. Patients were excluded if they had active perianal disease or if the severity of the disease could not be assessed either before or after the initiation of UPA treatment due to insufficient clinical or laboratory data. This study was approved by the institutional review board of Tokyo Yamate Medical Center (approval number 235) and conducted in accordance with the Declaration of Helsinki. An opt-out informed consent protocol was used for the use of participant data for research purposes.

### Data collection and assessment

Characteristics of the patients (including baseline renal and liver functions, and comorbidities assessed by the Charlson Comorbidity Index [CCI]), their treatment history, disease activity, and concurrent medications for IBD at UPA administration were manually reviewed. Medicines for IBD were documented as concomitant use if prescribed together with UPA and reviewed for their continuation. We also collected data on whether patients were treated as outpatients or inpatients. Drug adherence was assessed by reviewing prescription records in the electronic medical records to identify discrepancies in prescribed days and visit timings, and by confirming the absence of tracing reports from pharmacies regarding adherence issues. Efficacy was assessed with response and remission rates at weeks 8 and 16 for UC, and at week 12 for CD. We also analyzed the changes in serum C-reactive protein (CRP) and Albumin (Alb) levels. The allowable period for data usage was 10 days before and after each scheduled evaluation date. The median error in measurement dates was −2 days (IQR: −7, 7). The treatment response in patients with UC was evaluated based on the changes in the Partial Mayo Score (PMS) from baseline. It was considered effective if the score decreased by 2 points or more, or remission if the score was 2 or less, with a rectal bleeding sub-score of 0 and all other sub-scores were less than 1. For CD, efficacy was determined by the Crohn’s Disease Activity Index (CDAI). An effective response was defined by a ≥70-point CDAI reduction or remission (CDAI ≤ 150). The assessment of disease severity was done retrospectively from the electronic medical record. For each patient, we reviewed their treatment outcomes up to 52 weeks after UPA administration. We assessed the maintenance of remission, additional treatments or surgery, final dose of UPA, and the rate of medication persistence. Any complications or adverse events were also documented, and the cardiovascular risk and adverse events were assessed by QRISK3 [9] and CTCAE version 5.0, respectively.

### Statistical analysis

Categorical variables are summarized as frequencies and percentages, and continuous variables are expressed as means ± standard deviations (SD). Sample distributions are confirmed by comparison between medians and interquartile ranges using Microsoft Excel (Microsoft Corporation, Redmond, WA). Categorical variables were compared by Fisher’s exact probability test using GraphPad Prism 8.0 (GraphPad Software Inc., San Diego, CA, USA). Median follow-up time and patient-year exposure were calculated by using Microsoft Excel. Missing data were not imputed. P-values of less than 0.05 were considered to be significant.

## Results

### Characteristics of patients

**Fig. 1:**
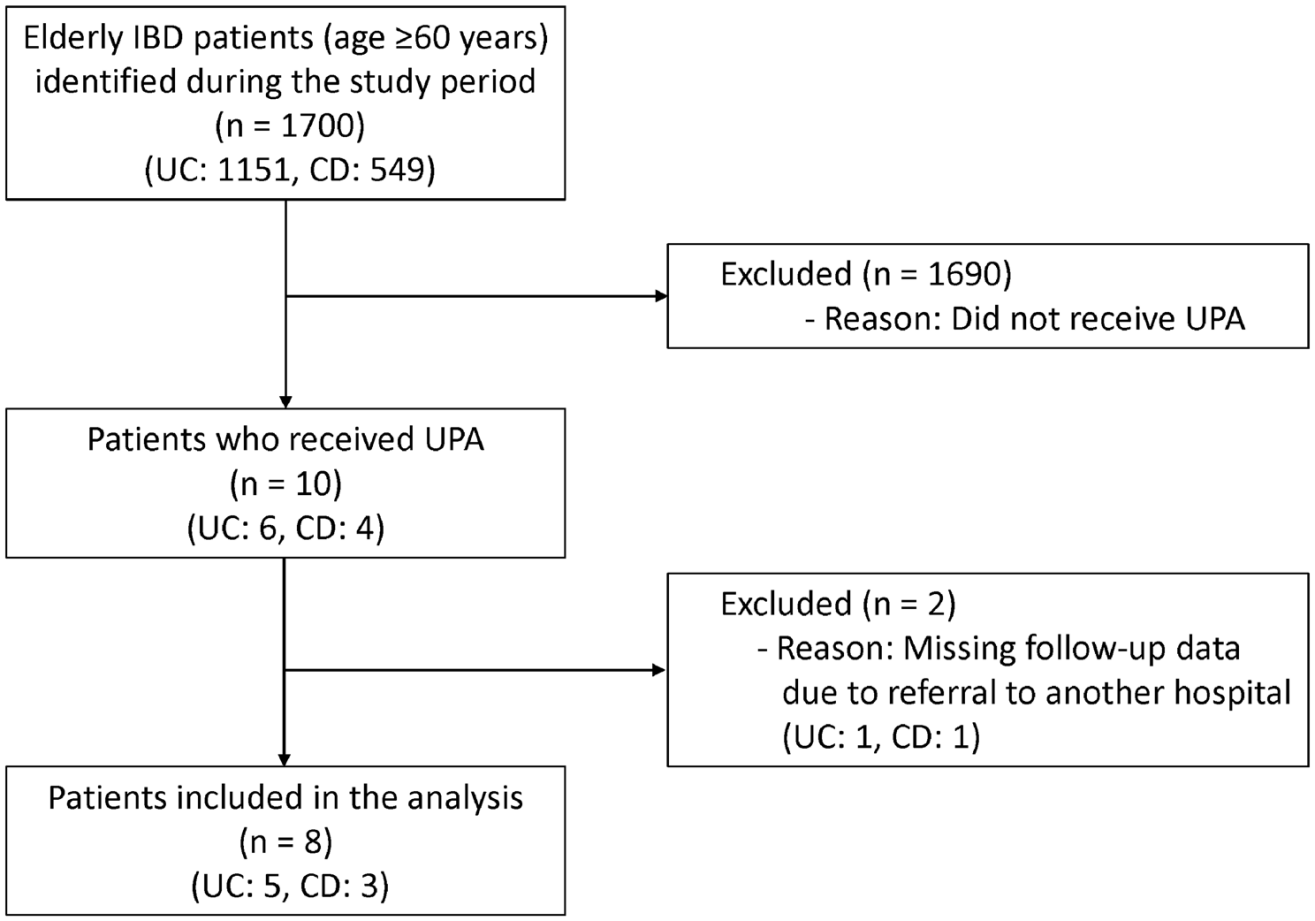
Flow diagram of patient selection

During the study period, 1700 elderly patients (aged ≥60 years) with IBD (1151 UC, 549 CD) were identified from the electronic medical records. Of these, 10 patients (6 UC, 4 CD) received UPA. We excluded 2 patients (1 UC, 1 CD) due to a lack of follow-up data after referral to another hospital. No patients were excluded due to active perianal disease. The remaining 8 patients (5 UC, 3 CD) were included in the final analysis (Fig. 1). The median age of the 8 patients was 63.5 years (interquartile range [IQR]: 61–70), and 75% of them were male. Median age at disease onset was 46 years (IQR: 33.5–56.8), and 2 UC patients presented with late-onset after 60 years old. Their median estimated 10-year risk of a cardiovascular event was 13.4% in QRISK3 (IQR: 9.8–21.9). Table 1 shows patient characteristics. Corticosteroid was co-administered in 3 UC patients at UPA induction, which was tapered off within 4 weeks in all cases. No CD patients received corticosteroids during the study period. All of the patients prescribed Mesalazine or Salazosulfapyridine at the initiation of UPA had been taking them continuously during the observation period. Seven patients (4 UC and 3 CD) were treated as outpatients, while one UC patient (Case 4) was hospitalized for acute severe UC, defined by Truelove and Witt’s criteria. Five patients (3 UC and 2 CD) had prior exposure to biologic agents; all but one UC patient had experienced at least one anti-TNF-α agent. UPA treatment was continued for over one year (≥52 weeks) in 3 UC patients (60%) and 2 CD patients (66.7%). One of the remaining UC patients had acute severe UC and underwent total colectomy at week 3 due to refractoriness to the treatment, and the other discontinued UPA at week 36 due to sustained anemia and liver injury, although colitis had been in remission since week 16. The remaining one CD patient has only been on UPA for 34 weeks but is currently in remission.

**Table 1:** Characteristics of the patients

	UC (*n* = 5)	CD (*n* = 3)	Total (*n* = 8)
Age (IQR)	65 (62, 69)	61 (60.5, 67)	63.5 (61, 70)
Male (%)	3 (60)	3 (100)	6 (75)
Age at onset (y) Median (IQR)	55 (45, 62)	34 (33, 40.5)	46 (33.5, 56.8)
Extension (%)	E1: 2(40) E3: 3(60)	L3: 3(100)	
Behavior (%)	NA	B2: 1(33.3) B2 + B3: 2(66.7)	
eGFR (IQR)	60.9 (55.5, 61)	57.9 (55.6, 74.6)	59.4 (54.9, 66.1)
Abnormal LFT (%)	2 (40)	1 (33.3)	3 (37.5)
CCI (IQR)	4 (4, 5)	3 (2.5, 3.5)	4 (3, 4.3)
Treatments before UPA initiation			
IFX	2 (40%)*	2 (66.7%)	4 (50%)
ADA	1 (20%)	0	1 (12.5%)
UST	1 (20%)*	0	1 (12.5%)
VDZ	1 (20%)*	0	1 (12.5%)
Thiopurine	2 (40%)^†^	3 (100%)	5 (62.5%)

IQR: interquartile range. E1: proctitis, E3: pan-colitis, B2: stricturing, B3: penetrating, eGFR: estimated glomerular filtration rate, LFT: Liver function test, CCI: Charlson comorbidity index. *One patient has experienced three biologics. ^†^Thiopurine was stopped upon initiation of UPA

### Clinical response and remission rates

**Fig. 2:**
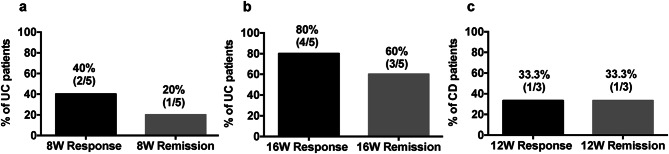
The clinical responses to UPA during induction phase in elderly patients with UC and CD. The rate of clinical response and remission in UC at week 8 (**a**), week 16 (**b**), and in CD at week 12 (**c**)

**Fig. 3:**
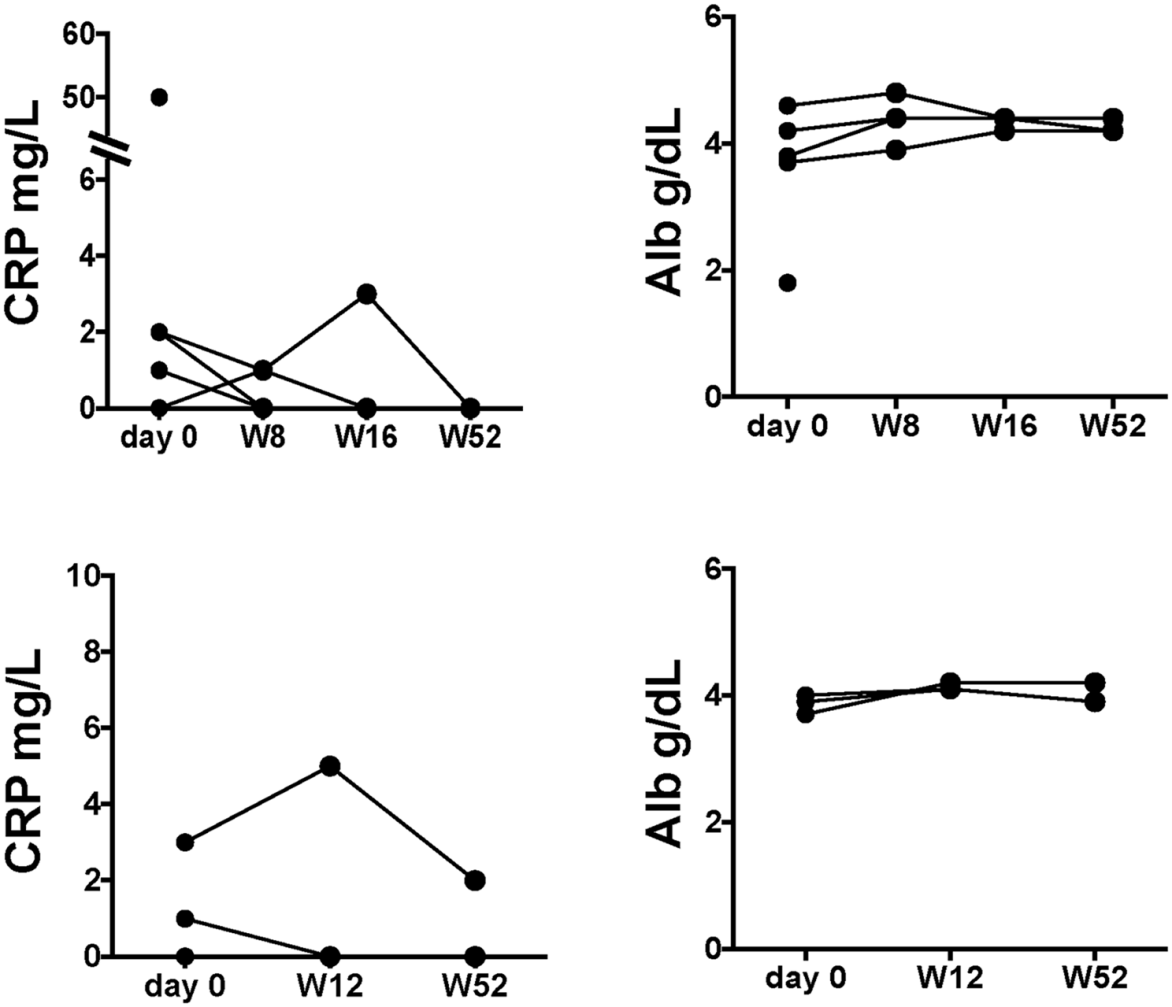
Changes in serum levels of CRP (mg/L) and Alb (g/dL) (top: UC, bottom: CD). One case each of UC and CD showed a temporal increase in serum CRP levels at weeks 16 and 12, respectively

The clinical response to UPA in UC patients was observed to be 40%, with a remission rate of 20% at week 8, which increased to 80% response and 60% remission at week 16, respectively (Fig. 2). On the other hand, the response and remission rates were both 33.3% at week 12 in patients with CD. Notably, the 2 CD patients we could follow for 52 weeks achieved remission afterward. Accordingly, the serum levels of CRP decreased by week 52 in the cases being followed for 52 weeks (Fig. 3). On the other hand, serum Alb levels did not change substantially during the course of UPA treatment. The final dose of UPA after induction therapy was 15 mg/day in 3 UC patients and 7.5 mg/day in the remaining UC patient. In patients with CD, the maintenance dose was 30 mg/day for 2 patients and 15 mg/day for one patient.

### Treatment outcomes and adverse events

**Fig. 4:**
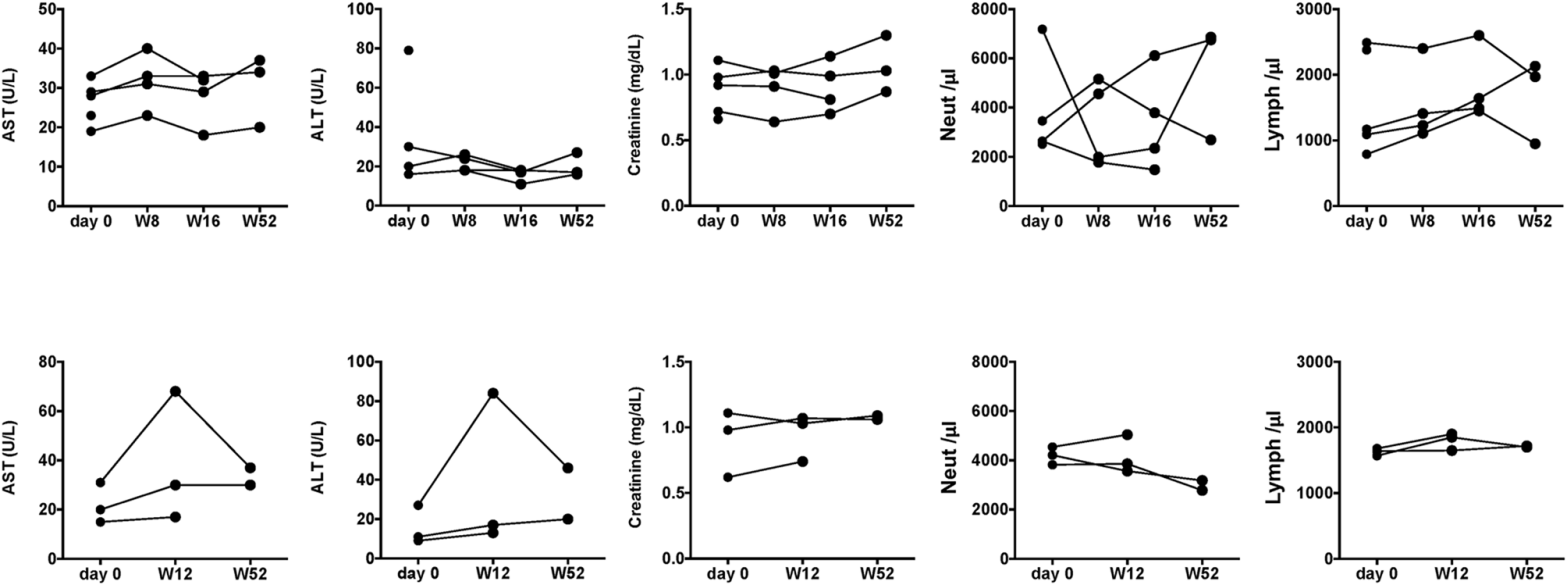
Changes in laboratory data (top: UC, bottom: CD). The kidney and the liver functions, and the number of blood cell elements were monitored

Table 2 presents the outcomes and adverse events for each case. Adverse events included nausea in one UC patient at week 4, and it resolved upon dose reduction from 30 to 15 mg/day. Another patient reported acne, stomatitis, and numbness of fingers, which improved with symptomatic management and graded CTCAE 1. One patient demonstrated increased serum CPK levels (200–300 IU/L) without subjective symptoms, and continued UPA treatment with watchful waiting (CTCAE 1). A transient elevation of transaminase levels was noted in one CD patient at week 12, which self-resolved and was considered unrelated to the therapy (CTCAE 1). During the median follow-up time of 14.1 months (95% CI 9.2, 24.4 months), adverse events of CTCAE grade 2 or higher were identified in two cases (Table 2). No severe infections, anaphylaxis, thromboembolisms, MACE, or malignancies were observed. In addition, no significant neutropenia, lymphopenia, or renal impairment that led to treatment cessation was detected (Fig. 4).

**Table 2:** Clinical course of individual patients

Case	IBD type	Age	Complications	Concomitant medications	Severity at day 0	Endoscopic activity	Severity at week 52	Follow up Endoscopy	AE	Final dose (mg/day)	Outcomes
1	UC	82	HTN, Atopic dermatitis	CS enema	PMS: 4	MES: 2	PMS: 2	MES: 1	Nausea CTCAE grade 2	15	Sustained remission
2	UC	69	HTN, DL	CS, 5ASA	PMS: 3	MES: 1	PMS: 1	NA	None	7.5	Sustained remission
3	UC	65	DL, Asthma	5ASA	PMS: 4	MES: 3	NA	NA	Anemia, Liver damage CTCAE grade 2	15	Discontinued at week 36
4	UC	62	T2DM, HTN	CS, 5ASA	PMS: 4	MES: 2	NA	NA	None	45	Underwent colectomy
5	UC	61	None	5ASA	PMS: 7	MES: 3	PMS: 1	MES: 1	Neuropathy, stomatitis, acne CTCAE grade 1	30	Sustained remission
6	CD	60	MDD	None	CDAI: 131	SES-CD: 5	NA	NA	None	30	Currently remission at week 27
7	CD	61	MDD	5ASA	CDAI: 170	SES-CD: 6	CDAI: 110	NA	None	15	Sustained remission
8	CD	73	DL, Renal insufficiency	SASP	CDAI: 181	SES-CD: 3	CDAI: 132	SES-CD: 0	None	30	Sustained remission

UC: Ulcerative Colitis, CD: Crohn’s Disease, HTN: Hypertension, DL: Dyslipidemia, T2DM: Type 2 Diabetes Mellitus, MDD: Major depressive disorder, CS: corticosteroid, 5ASA: 5-aminosalicylic acid, SASP: Salazosulfapyridine, PMS; Partial Mayo Score, CDAI: Crohn’s Disease Activity Index, AE: adverse event, CTCAE: Common Terminology Criteria for Adverse Events, NA: not available

## Discussion

This study investigated the response to UPA in elderly patients with IBD. In our cases, UPA demonstrated notable responses in both UC and CD, and the safety profile was relatively favorable, with limited adverse events and no thromboembolic events, MACE, infections, or malignancies reported.

Previous studies have demonstrated that 81.8% of patients with UC showed a therapeutic response to UPA and 57.6% of them achieved clinical remission at week 8 [10]. In patients with CD, it has been reported that 81.0% of patients showed a treatment response at week 12, and clinical remission was achieved in 77.8% of patients [10]. Another report evaluating the long-term effectiveness of UPA in CD patients for 52 weeks has demonstrated that clinical remission was achieved in 37.3% of patients receiving UPA 15 mg/day and 47.6% of those receiving 30 mg/day after the induction phase of 45 mg/day for 12 weeks [11]. Although these clinical trials of UPA have enrolled some elderly patients up to 75 years old, most of the participants were younger than 60 years old (mean age ranged 42.1 to 44.4 years), and the efficacy and safety data from a dedicated subgroup analysis in patients over 60 have not been reported [12]. Our results in elderly patients with IBD demonstrated that the short-term response was slightly lower than that in previous reports; however, the long-term response was consistent with previous data in both UC and CD individuals. Notably, the response rate at week 16 was higher than that of week 8 in 3 UC cases of this study, despite the dose of UPA being reduced from the induction dose at week 8. Therefore, it would be worth waiting until 16 weeks when we treat elderly UC patients with UPA, even if they do not show a clear response at week 8 in case the situation allows. Generally, the response rate of advanced therapies tends to be lower in patients with prior exposure to biologics than in those who are biologic-naïve. Although 63% of our patients had prior biologic experiences, the observed responses to UPA in both UC and CD seemed to be similar to biologic-naïve patients in previously reported data. Our finding is consistent with a recent meta-analysis that reports the effectiveness of UPA in highly refractory UC regardless of their history of treatment failure with biologics or other JAK inhibitors [13].

The safety of UPA was relatively favorable in our elderly patients, as most adverse events in this study were mild, with only one UC patient discontinued treatment due to persistent anemia. While the rate of anemia directly caused by UPA has been uncommon, the incidence of hematologic disorders has been reported to be about 1.56% [14]. The higher therapeutic doses of UPA for IBD than those for other diseases raise particular concerns about side effects, especially in elderly patients. Recent research on the safety of JAK inhibitors in elderly IBD patients reported a 23% risk of mild infections and a 3% risk of severe infections requiring hospitalization [15]. The Oral Rheumatoid Arthritis Trial (ORAL) Surveillance study demonstrated a significantly higher risk of MACE and malignancies in patients treated with tofacitinib compared to those receiving TNF inhibitors [16]. Consequently, the U.S. Food and Drug Administration (FDA) has issued a warning, based on the rationale that UPA may carry similar potential risks due to its shared mechanism of action. Compared to non-selective JAK inhibitors, more selective JAK1 inhibitors like UPA may offer a better safety profile, but long-term clinical data would still be needed. An analysis of UPA-related adverse events using the FDA Adverse Event Reporting System (FAERS) database showed that the most frequently reported adverse events in elderly patients were pulmonary embolism, cataracts, and sepsis [14]. In our cohort, neither pulmonary embolism nor cataracts were seen and no one experienced serious infectious diseases including herpes zoster. Geriatric syndromes (e.g., frailty, multi-morbidity) are highly prevalent and negatively impact outcomes in older patients with IBD, requiring systematic assessment and multidisciplinary intervention [17].

While this study provides some important information on the use of UPA in elderly IBD patients, there are several limitations due to the nature of a case series. First, the limited number of cases makes the findings difficult to generalize. Second, the retrospective data collection introduces potential information bias. The exclusion of 2 of 10 eligible patients due to missing follow-up data may have introduced selection bias and limited the representativeness of the cohort. Third, the lack of a control group prevents direct comparison with other advanced therapies. For instance, a previous report of a large meta-analysis has confirmed that elderly IBD patients on biologic therapy (primarily anti-TNF) had significantly increased risks of both infection (OR 3.48) and malignancy (OR 3.47) compared to younger patients [18]. Future head-to-head trials are warranted to clarify the optimal therapeutic positioning of UPA in elderly IBD patients. Finally, retrospective assessment of the medication adherence using prescription records may not guarantee 100% medication adherence, which may affect the results. There would be a need for a longer observation period to fully understand the safety profiles of UPA in patients with elderly IBD. Nevertheless, this report demonstrated substantial responses to UPA in elderly IBD patients without any major adverse events. Although the limited number of cases prevents us from drawing definitive conclusions, our findings provide a rationale for further large-scale, prospective data collection in the elderly IBD population.

## Data Availability

The data that support the findings of this study are not publicly available due to their containing information that could compromise the privacy of research participants but are available from the corresponding author M.F upon reasonable request.
